# Prostaglandins and Rheumatoid Arthritis

**DOI:** 10.1155/2012/239310

**Published:** 2012-11-07

**Authors:** Mohammad Javad Fattahi, Abbas Mirshafiey

**Affiliations:** Department of Immunology, School of Public Health, Tehran University of Medical Sciences, P.O. Box 6446, Tehran 14155, Iran

## Abstract

Rheumatoid arthritis (RA) is a chronic, autoimmune, and complex inflammatory disease leading to bone and cartilage destruction, whose cause remains obscure. Accumulation of genetic susceptibility, environmental factors, and dysregulated immune responses are necessary for mounting this self-reacting disease. Inflamed joints are infiltrated by a heterogeneous population of cellular and soluble mediators of the immune system, such as T cells, B cells, macrophages, cytokines, and prostaglandins (PGs). Prostaglandins are lipid inflammatory mediators derived from the arachidonic acid by multienzymatic reactions. They both sustain homeostatic mechanisms and mediate pathogenic processes, including the inflammatory reaction. They play both beneficial and harmful roles during inflammation, according to their site of action and the etiology of the inflammatory response. With respect to the role of PGs in inflammation, they can be effective mediators in the pathophysiology of RA. Thus the use of agonists or antagonists of PG receptors may be considered as a new therapeutic protocol in RA. In this paper, we try to elucidate the role of PGs in the immunopathology of RA.

## 1. Introduction

Rheumatoid arthritis (RA) is a complex autoimmune and progressive inflammatory disease that involves the joints and leads to their destruction. The prevalence of rheumatoid arthritis (RA) is 0.5%–1.0% in the general population worldwide [1, 2]. Females are nearly three times more likely than males to develop the disease and can start at any age, although the mean age at the onset is 40 to 60 years [3, 4]. The precise cause of rheumatoid arthritis is unknown; like other autoimmune diseases it arises from a variable combination of genetic susceptibility, environmental factors, and the inappropriate activation of the immune responses that eventually result in the clinical signs of arthritis [5]. Multiple genes are associated with disease susceptibility, with the HLA locus accounting for 30% to 50% of the overall genetic risk. Several risk loci have been recognized: HLA-DRB1, PTPN22, STAT4, CTLA4, RAD14 a region in 6q23, and the TRAF1/C5 locus [6–9]. Similarly, the mouse strains of DBA/1 and B10.Q have the I-Aq and I-Ar haplotypes and are highly susceptible to collagen-induced arthritis (CIA), as experimental models of RA [10, 11]. The important role of HLA-DR antigens is to present antigens to T lymphocytes, whereas the PTPN22 protein tyrosine phosphatase appears to have a potential function in the setting of T-cell and B-cell activation [12]. Smoking, the best-known environmental factor, in certain genetic context of HLA-DRB1 can trigger immunity to citrulline-modified proteins and this response, after several years, causes arthritis [13, 14]. The adaptive and innate immune responses in the synovial fluid are involved in the pathogenesis of RA. High levels of autoantibodies, including rheumatoid factors and anticitrullinated peptide antibodies, can be diagnosed before the onset of clinical arthritis [15]. Inflamed joint tissues are infiltrated by monocyte/macrophage, rheumatoid arthritis synovial fibroblast (RASF), T cells, and B cells. These cells release proinflammatory cytokines such as interleukin 1(IL-1), IL-17, and tumor necrosis factor *α*(TNF-*α*), that play important roles in progressive joint destruction and are closely associated with the production of small proinflammatory lipid mediators such as prostaglandins [16, 17]. 

## 2. Prostaglandins

Prostaglandins are small potent inflammatory mediators that are generated by the release of arachidonic acid (AA) from the membrane phospholipids by the phospholipase A2 (plpA2) family. Subsequently, cyclooxygenase (COX; prostaglandin endoperoxide H synthase; PGHS) and Prostaglandin synthase enzymes metabolize AA to prostaglandins including PGE_2_, PGF_2*α*_, PGD_2_, PGI_2_ (prostacyclin), and TXA_2_(thromboxane), that play pivotal roles in the modulation of physiological systems, such as CNS, and the inflammatory and immune responses [18, 19]. The cyclooxygenases are heme containing enzymes that have two major isoforms in mammals named COX-1 and COX-2. Although COX-1 and COX-2 have about 60% homology at the amino acid level and catalyze the same reactions, they have different patterns of expression and are encoded by different genes [20, 21]. COX-1 is constitutively expressed in many tissues and is responsible for the physiological function of PGs and thus is known as a “housekeeping” enzyme whereas COX-2 expression is induced by inflammatory mediators like cytokines, growth factors, and bacterial endotoxins [21, 22]. Traditional NSAIDs (nonsteroidal anti-inflammatory drugs), that have antipyretic, analgesic, and anti-inflammatory properties, inhibit both COX-1 and COX-2 and are associated with side effects such as gastrointestinal bleeding due to the suppression of both COX isozymes. The recently developed COX-2-selective inhibitors retain effectiveness in reducing inflammation and pain in rheumatoid and osteoarthritis but have a lower incidence of gastrointestinal side effects [23]. Prostaglandins use G-protein coupled receptors (GPCRs) for exerting their functions. The prostaglandins receptors subfamilies include DP, EP1-4, FP, IP, and TP which bind to PGD_2_, PGE_2_, PGF_2*α*_, PGI_2_, and TXA_2_, respectively [24]. Recently, another PGD_2_ receptor, the chemoattractant receptor homologous molecule expressed on Th2 cells (CRTH2), was identified [25]. The prostaglandins (PGs) found at elevated levels in the synovial fluid and the synovial membrane are considered to play a pivotal role in the development of vasodilatation, fluid extravasation, and pain in synovial tissues. Moreover, there is an increasing evidence that PGs (especially prostaglandin E_2_) are mediators involved in complex interactions leading to the development of erosions of the articular cartilage and the juxta-articular bone [26]. PG synthesis inhibitors (COX-2 inhibitors and NSAIDs) are widely used in the treatment of RA [27].

## 3. Prostaglandins and Rheumatoid Arthritis

### 3.1. PGD_2_ and RA

Prostaglandin D synthase is responsible for the generation of the PGD_2_ and J series. This enzyme has two isoforms including the lipocalin (brain) type PGDS (L-PGDS) which is responsible for PGD_2_ biosynthesis in the CNS where it plays a central role in the regulation of sleep and the hematopoietic PGDS (h-PGDS) which is also known as “spleen type” PGD_2_ synthase [28, 29]. PGD_2_ regulates multiple physiologic and pathologic processes, such as sleep, nociception, vasodilation, bronchoconstriction, and bone metabolism. PGD_2_ has anti-inflammatory effects in several models of inflammation [30, 31]. It exerts its function by binding two receptors, DP1 and CRTH2. DP1 activation by PDG_2_ causes the elevation of intracellular cAMP level which is typically associated with damping of the cellular effector function, whereas the CRTH2/DP2 receptor stimulation leads to the elevation of intracellular Ca^+2^. DP2 activation by PGD_2_ induces TH2 cytokines production, their migration, and enhancing their adhesiveness to endothelial surfaces [29]. On the other hand, when PGD_2_ is produced in a large amount for activating PPAR-*γ*, it will be able to inhibit T-lymphocyte proliferation and, consequently, the inflammatory response. In contrast, if only nanomolar concentrations of PGD_2_ and its metabolites are produced, the PGs may be expected to activate T lymphocytes [25, 32, 33]. In synovial fluid, PGD_2_ abundantly is released by chondrocytes [33], osteoclasts [34], synovial fibroblast, and mast cells [35, 36]. It inhibits chondrocyte apoptosis [37], stimulates chondrogenic differentiation, enhances the expression of collagen type II and aggrecan [38], prevents IL-1-induced generation of MMP-1 (metalloproteinase-1) and MMP-13 by chondrocytes through the DP1/cAMP/PKA signaling pathway, indicating that PGD_2_ may contribute to the cartilage maintenance and integrity [39, 40]. PGD_2_ is readily dehydrated and generates PGs of the J series, such as PGJ_2_, *δ*12-PGJ_2_, and 15-deoxy-*δ*12,14-PGJ_2_ (15d-PGJ_2_). The 15d-PGJ_2_ was identified as a ligand for peroxisome proliferator activated receptor-*γ* (PPAR-*γ*) which enhances the differentiation of adipocytes and trophoblasts [41, 42] and implicates as a mediator of many anti-inflammatory effects of PGD_2_ [43]. 15d-PGJ_2_ is released by articular chondrocytes and diagnosed in synovial fluid RA patients. It enhances chondrocyte apoptosis in a dose- and time-dependent manner by a PPAR*γ*-dependent pathway [44]. PGD_2_ and its metabolite, 15d-PGJ_2_, may have chondroprotective effects. For instance, they counteract the induction of matrix metalloproteinases in cytokine-activated chondrocytes, which play an important role in cartilage degradation [39, 45]. The 15d-PGJ_2_ inhibits the production of several inflammatory mediators by monocytes/macrophages. It blocks nitric oxide (NO) production as well as proteoglycan degradation [45–48]. 15d-PGJ_2_ also inhibits apoptosis of human primary chondrocytes induced by the NF-*κ*B inhibitor (Bay 11–7085) [37, 49].

### 3.2. PGE_2_ and RA

PGE_2_ is the major PG that is generated by chondrocytes and synovial fibroblasts; the biosynthesis can be enhanced by proinflammatory cytokines such as IL-1*β*, TNF-*α*, and trauma [50]. Prostaglandin E synthase (PGES) converts COX-derived PGH2 to PGE_2_ [51], a potent lipid mediator, that regulates a broad range of physiological activities in the immune and the other biological systems such as cardiovascular, endocrine, gastrointestinal, neural, pulmonary, reproductive, and visual systems [52]. Three different forms of the PGESs have been identified, microsomal PGES-1,2 (mPGES-1,2) and cytosolic PGES(cPGES, p23). mPGES-1 is preferentially linked with COX-2 and is induced in response to various stimuli [53]. Glutathione-independent mPGES-2 is a unique PGES that is constitutively expressed and coupled with both COXs in the production of the PGE_2_ involved in both tissue homeostasis and disease [30, 54]. The cPGES is constitutively expressed and to be preferentially coupled to COX-1 than COX-2 and its expression is not affected by proinflammatory stimuli [55]. Of the three PGES isozymes, mPGES-1 is upregulated in synovial fluid in active RA and is minimally expressed in inactive RA [56]. The mPGES-1 induction is coordinated with COX-2 expression under inflammatory conditions in different cells and tissues as well as RA synovium. Some of selective COX-2 inhibitors may cause cardiovascular side effects in RA patients due to simultaneous decrease in production of PGE2 and antithrombotic PGI2. In order to reduce this side effect selective inhibition of mPGES-1 derived PGE2 production will be an desirable therapeutic alternative [57, 58]. PGE_2_ exerts its diverse roles by acting on a group of rhodopsin-like 7-transmembrane-spanning GPCRs: EP1, EP2, EP3, and EP4. The EP subtypes show differences in binding affinity, signal transduction, tissue localization, and regulation of expression. The Ep3 and EP4 are the most abundant of the EP receptors and their binding affinity to PGE2 is higher than EP1 and EP2 receptors [59, 60]. EP receptors link to different intracellular signaling molecules that mediate the effects of receptor activation on cell function. EP2 and EP4 receptors couple to a Gs-type G-protein that activate adenylate cyclase, increasing intracellular cAMP. EP1 links to Gq and activates phosphatidylinositol metabolism leading to mobilization of intracellular free calcium. EP3 receptor can couple to Gi or G12 for elevation of intracellular Ca^2+^, inhibition of cAMP generation, and activation of the small G-protein Rho [19, 60]. 

The knock-out mouse studies revealed that PGE_2_ can exert both proinflammatory and anti-inflammatory responses, depending on receptor subtype, cell population, context of activation, and receptor gene expression in tissues [61]. Using mice deficient in the EP subtypes, Honda et al. found that the simultaneous inhibition of EP2 and EP4 significantly decreased the arthritic score in CIA. Loss or inhibition of a single EP does not affect the extent of CIA [62]. Stock et al. using EP1-deficient(EP1-/-) mice showed a reduced stretching response following administration of acetic acid or 2-phenyl-1 benzoquinone (PBQ) suggesting that the central hyperalgesic effects of PGE_2_ are mediated by spinal EP1 receptors [63]. Minami et al. observed that the PGE_2_-induced hyperalgesia is mediated by the EP3 receptor at lower doses and by the EP2 receptor at higher doses [64]. Yao et al. showed that Th1 and IL-23-dependent Th17 differentiation is promoted by PGE_2_-EP4 signaling in DCs and T cells [65]. PGE_2_/EP4 plays a proinflammatory role in the pathogenesis of rheumatoid arthritis as in CIA, the homozygous deletion of EP4(EP4-/-) receptor but not the EP1, EP2, or EP3 receptors, which led to decrease in incidence and severity of disease [66]. The EP1 downregulates the expression of COX-2 in a concentration-dependent approach through a receptor activation-independent pathway. The reduction in COX-2 protein occurs due to the enhancement of substrate-independent COX-2 proteolysis because EP1 facilitates COX-2 ubiquitination via an unknown E3 ligase. This suggests a new role for the EP1 in resolving inflammation [67]. It has been shown that chondrocyte apoptosis is induced by PGE_2_ binding to EP2 or EP4 receptors [68]. This is dependent on the activation of the cell, since PGE_2_ enhances apoptosis in resting mature T cells, whereas it protects T cells from T-cell receptor mediated activation-induced apoptosis [69, 70]. Aoyama et al. found the dominant expression of EP2 receptors in human articular cartilage and cultured chondrocytes, whereas EP1 and EP4 receptor expression was not significantly increased [71]. Otsuka et al. showed that PGE2 signal via EP2 not only has an anti-inflammatory property but also promotes chondrocyte proliferation and the regeneration of articular cartilage. Attur et al. reported that the catabolic activities of PGE_2_ is mediated by PGE_2_-EP4 signaling in OA cartilage. PGE_2_-EP4 induces matrix metalloproteinase production and type II collagen degradation. PGE_2_ through EP4 receptor shows a potent antianabolic effect on human adult articular cartilage in vitro via the suppression of proteoglycan biosynthesis, which suggests EP4 receptor antagonist could be as a potential therapeutic agent for the treatment of osteoarthritis and RA [72, 73]. Clark et al. also found that a selective EP4 antagonist reduces COX-2-dependent arthritic inflammation and pain (NSAID-like activity). This reagent is well tolerated by the gastrointestinal tract and, unlike COX-2 inhibitors, it does not inhibit PGI2 and may be cardioprotective [74]. PGE_2_ has also inhibitory effects on NF-*κ*B through ERK-dependent and -independent pathways in RASF, key mediators of RA inflammation, and cartilage erosion. This process may paradoxically inhibit the action of inflammatory cytokines and may participate in the resolution phase of inflammation to prevent cartilage degradation in arthritis [75]. 

### 3.3. PGI_2_ and RA

Prostacyclin (PGI_2_) the main PG produced by vascular endothelial cells exerts its functions through a seven-transmembrane-spanning GPCR, known as the IP receptor. Both cyclooxygenase enzymes (COX-1/2) convert AA into the prostaglandin precursor PGH_2_, which is subsequently converted into PGI_2_ via prostacyclin synthase (PGIS), a member of cytochrome P450 superfamily [76, 77]. The IP receptor is coupled predominately to a Gs subunit (and in some circumstances with Gi- and Gq-dependent pathways) and the G-protein leads to an increase in the cAMP level and this signaling pathway is responsible for vasodilatory and antithrombotic effects of prostacyclin [78–80]. PGI_2_ may also signal through the PPAR-*γ* pathway [81]. Prostacyclin plays a regulatory role within the cardiovascular system. It has been found that the IP receptor signaling by enhancing Th2-cell production of the anti-inflammatory cytokine IL-10 inhibits Th2 mediated allergic inflammatory responses [19, 82]. PGI_2_ is the most frequent prostaglandin in synovial fluid of patients with RA [83]. In rheumatoid arthritis PGI_2_ acts as a proinflammatory lipid mediator. IP receptor antagonists inhibit experimental hyperalgesia, edema, and osteoarthritis in the rat, indicating that prostacyclin plays an important role in these pathological conditions. In CIA, IP receptor-deficient mice showed a significant decrease in arthritic score in spite of anticollagen antibodies and complement activation similar to wild-type mice. In addition, the administration of the IP antagonist in this model also reduced the symptoms (NSAID-like efficacy) [84, 85].

### 3.4. PGF_2*α*_ and RA

Prostaglandin F_2*α*_(PGF_2*α*_) is biosynthesized from PGH_2_ and other PGs (PGE_2_, PGD_2_) by three enzymes, PGH 9-, 11-endoperoxide reductase, PGE 9-ketoreductase, and PGD 11-ketoreductase, respectively [86]. It exerts its biological functions by binding to a prostanoid receptor FP which has two differentially spliced variants (FP_A_, FP_B_). The FP receptor couples with the G_q_ protein for increasing the inositol phosphate accumulation, protein kinase C (PKC) activation, and intracellular calcium release [87]. In addition, stimulation of FP receptor leads to activation of G-protein Rho via a Gq-independent process, resulting in cytoskeleton rearrangement [88]. The FP receptor is the least selective of the prostaglandin receptors in binding the principal endogenous prostaglandins, binding both PGD_2_ and PGE_2_ at nanomolar concentrations [19]. PGF_2*α*_ has a pivotal role in the reproductive system, renal function, contraction of arteries, myocardial dysfunction, and regulation of intraocular pressure and pain [89–93]. Basu showed that the oxidative metabolism of arachidonic acid through both enzymatic (cyclooxygenase) and nonenzymatic (free radical) pathways is engaged in endotoxin-induced inflammation in pigs as indicated by the significantly increased formation of F_2_-isoprostane and PGF_2*α*_ metabolite in plasma [94]. They also showed that the measurement of F_2_-isoprostanes in body fluids provides a reliable analytical tool to study oxidative stress-related diseases and experimental inflammatory conditions [95]. High levels of both free radical mediated F_2_-isoprostanes and the cyclooxygenase derived PGF_2*α*_ metabolite were diagnosed in blood and synovial fluid from patients with various rheumatic diseases such as RA and OA that shows both oxidative injury and inflammation play a role in various degrees in chronic inflammatory conditions [96]. The arising role of PGF_2*α*_ in inflammatory reactions opens the unique opportunities for designing the new anti-inflammatory drugs [61].

## 4. Conclusion

Elevated levels of prostaglandins have been diagnosed in the synovial fluid and synovial membrane of RA patients. In the inflamed joints PGs play pivotal roles through complex interactions with leukocytes and other cells. They can induce both pro- and anti-inflammatory responses, depending on the receptor subtype, cell population, context of activation, and the receptor gene expression in tissues. The role of prostaglandins in the metabolism of articular cartilage is still controversial. Some studies show that prostaglandins contribute to the destruction of articular cartilage by degrading cartilage ECM, while others found that they induce chondrogenesis and terminal differentiation. The different biological roles attributed to these lipid mediators are a direct indication of the molecular complexity of prostaglandins and their exclusive cognate receptors. Mice deficient in individual PGs receptors and combinations of these receptors will allow the investigation of the role of these receptors and their ligands in various models of inflammatory diseases such as RA. The most important therapies for RA should both inhibit inflammation and activate resolution. A broad spectrum of different enzyme inhibitors and receptor antagonists has been studied, showing a variety of effects on the course of the disease. Thus, it seems that the pharmacological intervention to modulate the release of lipid mediators will be important to improve the patient outcomes. The research efforts of recent years, however, contribute to a better understanding of the pathophysiological impact of lipid mediators in inflammatory disorders and provide new therapeutic approaches.

## References

[1] Lawrence RC, Helmick CG, Arnett FC (1998). Estimates of the prevalence of arthritis and selected musculoskeletal disorders in the United States. *Arthritis & Rheumatism*.

[2] Silman AJ, Pearson JE (2002). Epidemiology and genetics of rheumatoid arthritis. *Arthritis Research*.

[3] Smolen JS, Steiner G (2003). Therapeutic strategies for rheumatoid arthritis. *Nature Reviews Drug Discovery*.

[4] O'Dell JR, Smolen JS, Aletaha D, Robinson DR, Saint Clair EW, Stone JH (2010). Rheumatoid arthritis. *A Clinician's Pearls and Myths in Rheumatology*.

[5] Huber LC, Distler O, Tarner I, Gay RE, Gay S, Pap T (2006). Synovial fibroblasts: key players in rheumatoid arthritis. *Rheumatology*.

[6] Imboden JB (2009). The immunopathogenesis of rheumatoid arthritis. *Annual Review of Pathology*.

[7] De Vries R (2011). Genetics of rheumatoid arthritis: time for a change!. *Current Opinion in Rheumatology*.

[8] Eyre S, Barton A, Shephard N (2004). Investigation of susceptibility loci identified in the UK rheumatoid arthritis whole-genome scan in a further series of 217 UK affected sibling pairs. *Arthritis and Rheumatism*.

[9] Jawaheer D, Seldin MF, Amos CI (2003). Screening the genome for rheumatoid arthritis susceptibility genes: a replication study and combined analysis of 512 multicase families. *Arthritis and Rheumatism*.

[10] Doncarli A, Chiocchia G, Stasiuk LM (1999). A recurrent valpha17/vbeta10 TCR-expressing T cell clone is involved in the pathogenicity of collagen-induced arthritis in DBA/1 mice. *European Journal of Immunology*.

[11] Osman GE, Toda M, Kanagawa O, Hood LE (1993). Characterization of the T cell receptor repertoire causing collagen arthritis in mice. *Journal of Experimental Medicine*.

[12] Källberg H, Padyukov L, Plenge RM (2007). Gene-gene and gene-environment interactions involving HLA-BRB1, PTPN22, and smoking in two subsets of rheumatoid arthritis. *American Journal of Human Genetics*.

[13] Klareskog L, Padyukov L, Lorentzen J, Alfredsson L (2006). Mechanisms of disease: genetic susceptibility and environmental triggers in the development of rheumatoid arthritis. *Nature Clinical Practice Rheumatology*.

[14] Tobón GJ, Youinou P, Saraux A (2010). The environment, geo-epidemiology, and autoimmune disease: rheumatoid arthritis. *Autoimmunity Reviews*.

[15] Nielen MMJ, Van Schaardenburg D, Reesink HW (2004). Specific autoantibodies precede the symptoms of rheumatoid arthritis: a study of serial measurements in blood donors. *Arthritis and Rheumatism*.

[16] Kuligowska M, Odrowaz-Sypniewska G (2004). Role of interleukin-17 in cartilage and bone destruction in rheumatoid arthritis. *Ortopedia, Traumatologia, Rehabilitacja*.

[17] Zwerina J, Redlich K, Schett G, Smolen JS (2005). Pathogenesis of rheumatoid arthritis: targeting cytokines. *Annals of the New York Academy of Sciences*.

[18] Shimizu T (2009). Lipid mediators in health and disease: enzymes and receptors as therapeutic targets for the regulation of immunity and inflammation. *Annual Review of Pharmacology and Toxicology*.

[19] Hata AN, Breyer RM (2004). Pharmacology and signaling of prostaglandin receptors: multiple roles in inflammation and immune modulation. *Pharmacology and Therapeutics*.

[20] Milatovic D, Montine TJ, Aschner M (2011). Prostanoid signaling: dual role for prostaglandin E2 in neurotoxicity. *NeuroToxicology*.

[21] Rouzer CA, Marnett LJ (2009). Cyclooxygenases: structural and functional insights. *Journal of Lipid Research*.

[22] Hayashi S, Ueno N, Murase A, Nakagawa Y, Takada J (2012). Novel acid-type cyclooxygenase-2 inhibitors: design, synthesis, and structure-activity relationship for anti-inflammatory drug. *European Journal of Medicinal Chemistry*.

[23] FitzGerald GA, Patrono C (2001). The coxibs, selective inhibitors of cyclooxygenase-2. *The New England Journal of Medicine*.

[24] Breyer RM, Bagdassarian CK, Myers SA, Breyer MD (2001). Prostanoid receptors: subtypes and signaling. *Annual Review of Pharmacology and Toxicology*.

[25] Hirai H, Tanaka K, Yoshie O (2001). Prostaglandin D2 selectively induces chemotaxis in T helper type 2 cells, eosinophils, and basophils via seven-transmembrane receptor CRTH2. *Journal of Experimental Medicine*.

[26] Crofford LJ, Wilder RL, Ristimaki AP (1994). Cyclooxygenase-1 and -2 expression in rheumatoid synovial tissues. Effects of interleukin-1*β*, phorbol ester, and corticosteroids. *Journal of Clinical Investigation*.

[27] Pulichino AM, Rowland S, Wu T (2006). Prostacyclin antagonism reduces pain and inflammation in rodent models of hyperalgesia and chronic arthritis. *Journal of Pharmacology and Experimental Therapeutics*.

[28] Saito S, Tsuda H, Michimata T (2002). Prostaglandin D2 and reproduction. *American Journal of Reproductive Immunology*.

[29] Khanapure SP, Garvey DS, Janero DR, Letts LG (2007). Eicosanoids in inflammation: biosynthesis, pharmacology, and therapeutic frontiers. *Current Topics in Medicinal Chemistry*.

[30] Murakami M, Nakashima K, Kamei D (2003). Cellular prostaglandin E2 production by membrane-bound prostaglandin E synthase-2 via both cyclooxygenases-1 and -2. *The Journal of Biological Chemistry*.

[31] Gilroy DW, Colville-Nash PR, Willis D, Chivers J, Paul-Clark MJ, Willoughby DA (1999). Inducible cyclooxygenase may have anti-inflammatory properties. *Nature Medicine*.

[32] Tanaka K, Hirai H, Takano S, Nakamura M, Nagata K (2004). Effects of prostaglandin D2 on helper T cell functions. *Biochemical and Biophysical Research Communications*.

[33] Egg D (1984). Concentration of prostaglandins D2, E2, F(2*α*), 6-keto-F(1*α*) and thromboxane B2 in synovial fluid from patients with inflammatory joint disorders and osteoarthritis. *Zeitschrift fur Rheumatologie*.

[34] Gallant MA, Samadfam R, Hackett JA, Antoniou J, Parent JL, De Brum-Fernandes AJ (2005). Production of prostaglandin D2 by human osteoblasts and modulation of osteoprotegerin, RANKL, and cellular migration by DP and CRTH2 receptors. *Journal of Bone and Mineral Research*.

[35] Pietila P, Moilanen E, Seppala E (1984). Differences in the production of arachidonic acid metabolites between healthy and rheumatic synovial fibroblasts in vitro. A preliminary study. *Scandinavian Journal of Rheumatology*.

[36] De Paulis A, Marinò I, Ciccarelli A (1996). Human synovial mast cells: I. Ultrastructural in situ and in vitro immunologic characterization. *Arthritis and Rheumatism*.

[37] Relić B, Benoit V, Franchimont N (2004). 15-Deoxy-Δ12,14-prostaglandin J2 inhibits bay 11-7085-induced sustained extracellular signal-regulated kinase phosphorylation and apoptosis in human articular chondrocytes and synovial fibroblasts. *The Journal of Biological Chemistry*.

[38] Jakob M, Démarteau O, Suetterlin R, Heberer M, Martin I (2004). Chondrogenesis of expanded adult human articular chondrocytes is enhanced by specific prostaglandins. *Rheumatology*.

[39] Zayed N, Afif H, Chabane N (2008). Inhibition of interleukin-1*β*-induced matrix metalloproteinases 1 and 13 production in human osteoarthritic chondrocytes by prostaglandin D 2. *Arthritis and Rheumatism*.

[40] Zayed N, Li X, Chabane N (2008). Increased expression of lipocalin-type prostaglandin D2 synthase in osteoarthritic cartilage. *Arthritis Research and Therapy*.

[41] Schaiff WT, Carlson MG, Smith SD, Levy R, Nelson DM, Sadovsky Y (2000). Peroxisome proliferator-activated receptor-*γ* modulates differentiation of human trophoblast in a ligand-specific manner. *Journal of Clinical Endocrinology and Metabolism*.

[42] Forman BM, Tontonoz P, Chen J, Brun RP, Spiegelman BM, Evans RM (1995). 15-deoxy-Δ12, 14-prostaglandin J2 is a ligand for the adipocyte determination factor PPAR*γ*. *Cell*.

[43] Trivedi SG, Newson J, Rajakariar R (2006). Essential role for hematopoietic prostaglandin D2 synthase in the control of delayed type hypersensitivity. *Proceedings of the National Academy of Sciences of the United States of America*.

[44] Shan ZZ, Masuko-Hongo K, Dai SM, Nakamura H, Kato T, Nishioka K (2004). A potential role of 15-deoxy-Δ12,14-prostaglandin J2 for induction of human articular chondrocyte apoptosis in arthritis. *The Journal of Biological Chemistry*.

[45] Fahmi H, Di Battista JA, Pelletier JP, Mineau F, Ranger P, Martel-Pelletier J (2001). Peroxisome proliferator—activated receptor gamma activators inhibit interleukin-1beta-induced nitric oxide and matrix metalloproteinase 13 production in human chondrocytes. *Arthritis & Rheumatism*.

[46] Ricote M, Li AC, Willson TM, Kelly CJ, Glass CK (1998). The peroxisome proliferator-activated receptor-*γ* is a negative regulator of macrophage activation. *Nature*.

[47] Jiang C, Ting AT, Seed B (1998). PPAR-*γ* agonists inhibit production of monocyte inflammatory cytokines. *Nature*.

[48] Bordji K, Grillasca JP, Gouze JN (2000). Evidence for the presence of peroxisome proliferator-activated receptor (PPAR) *α* and *γ* and retinoid Z receptor in cartilage. PPAr*γ* activation modulates the effects of interleukin-1*β* on rat chondrocytes. *The Journal of Biological Chemistry*.

[49] Zhu F, Wang P, Kontrogianni-Konstantopoulos A, Konstantopoulos K (2010). Prostaglandin (PG)D(2) and 15-deoxy-Delta(12,14)-PGJ(2), but not PGE(2), mediate shear-induced chondrocyte apoptosis via protein kinase A-dependent regulation of polo-like kinases. *Cell Death and Differentiation*.

[50] Martel-Pelletier J, Pelletier JP, Fahmi H (2003). Cyclooxygenase-2 and prostaglandins in articular tissues. *Seminars in Arthritis and Rheumatism*.

[51] Claveau D, Sirinyan M, Guay J (2003). Microsomal prostaglandin E synthase-1 is a major terminal synthase that is selectively up-regulated during cyclooxygenase-2-dependent prostaglandin E2 production in the rat adjuvant-induced arthritis model. *Journal of Immunology*.

[52] Akaogi J, Nozaki T, Satoh M, Yamada H (2006). Role of PGE2 and EP receptors in the pathogenesis of rheumatoid arthritis and as a novel therapeutic strategy. *Endocrine, Metabolic and Immune Disorders - Drug Targets*.

[53] Gudis K, Sakamoto C (2005). The role of cyclooxygenase in gastric mucosal protection. *Digestive Diseases and Sciences*.

[54] Tanikawa N, Ohmiya Y, Ohkubo H (2002). Identification and characterization of a novel type of membrane-associated prostaglandin E synthase. *Biochemical and Biophysical Research Communications*.

[55] Tanioka T, Nakatani Y, Semmyo N, Murakami M, Kudo I (2000). Molecular identification of cytosolic prostaglandin E2 synthase that is functionally coupled with cyclooxygenase-1 in immediate prostaglandin E2 biosynthesis. *The Journal of Biological Chemistry*.

[56] Sano H (2011). The role of lipid mediators in the pathogenesis of rheumatoid arthritis. *Inflamation and Regeneration*.

[57] Bombardier C, Laine L, Reicin A (2000). Comparison of upper gastrointestinal toxicity of rofecoxib and naproxen in patients with rheumatoid arthritis. *The New England Journal of Medicine*.

[58] McAdam BF, Catella-Lawson F, Mardini IA, Kapoor S, Lawson JA, FitzGerald : GA (1999). Systemic biosynthesis of prostacyclin by cyclooxygenase (COX)-2: the human pharmacology of a selective inhibitor of COX-2. *Proceedings of the National Academy of Sciences of the United States of America*.

[59] Abramovitz M, Adam M, Boie Y (2000). The utilization of recombinant prostanoid receptors to determine the affinities and selectivities of prostaglandins and related analogs. *Biochimica et Biophysica Acta*.

[60] Sugimoto Y, Narumiya S (2007). Prostaglandin E receptors. *The Journal of Biological Chemistry*.

[61] Ricciotti E, FitzGerald GA (2011). Prostaglandins and inflammation. *Arteriosclerosis, Thrombosis, and Vascular Biology*.

[62] Honda T, Segi-Nishida E, Miyachi Y, Narumiya S (2006). Prostacyclin-IP signaling and prostaglandin E2-EP2/EP4 signaling both mediate joint inflammation in mouse collagen-induced arthritis. *Journal of Experimental Medicine*.

[63] Stock JL, Shinjo K, Burkhardt J (2001). The prostaglandin E2 EP1 receptor mediates pain perception and regulates blood pressure. *Journal of Clinical Investigation*.

[64] Minami T, Nakano H, Kobayashi T (2001). Characterization of EP receptor subtypes responsible for prostaglandin E2-induced pain responses by use of EP1 and EP3 receptor knockout mice. *British Journal of Pharmacology*.

[65] Yao C, Sakata D, Esaki Y (2009). Prostaglandin E2-EP4 signaling promotes immune inflammation through TH1 cell differentiation and TH17 cell expansion. *Nature Medicine*.

[66] McCoy JM, Wicks JR, Audoly LP (2002). The role of prostaglandin E2 receptors in the pathogenesis of rheumatoid arthritis. *Journal of Clinical Investigation*.

[67] Haddad A, Flint-Ashtamker G, Minzel W, Sood R, Rimon G, Barki-Harrington L (2012). Prostaglandin EP1 receptor down-regulates expression of cyclooxygenase-2 by facilitating its proteasomal degradation. *The Journal of Biological Chemistry*.

[68] Miwa M, Saura R, Hirata S, Hayashi Y, Mizuno K, Itoh H (2000). Induction of apoptosis in bovine articular chondrocyte by prostaglandin E2 through cAMP-dependent pathway. *Osteoarthritis and Cartilage*.

[69] Pica F, Franzese O, D’Onofrio C, Bonmassar E, Favalli C, Garaci E (1996). Prostaglandin E2 induces apoptosis in resting immature and mature human lymphocytes: a c-Myc-dependent and Bcl-2-independent associated pathway. *Journal of Pharmacology and Experimental Therapeutics*.

[70] Porter BO, Malek TR (1999). Prostaglandin E2 inhibits T cell activation-induced apoptosis and Fas-mediated cellular cytotoxicity by blockade of Fas-ligand induction. *European Journal of Immunology*.

[71] Aoyama T, Liang B, Okamoto T (2005). PGE2 signal through EP2 promotes the growth of articular chondrocytes. *Journal of Bone and Mineral Research*.

[72] Attur M, Al-Mussawir HE, Patel J (2008). Prostaglandin E2 exerts catabolic effects in osteoarthritis cartilage: evidence for signaling via the EP4 receptor. *Journal of Immunology*.

[73] Otsuka S, Aoyama T, Furu M (2009). PGE2 signal via EP2 receptors evoked by a selective agonist enhances regeneration of injured articular cartilage. *Osteoarthritis and Cartilage*.

[74] Clark P, Rowland SE, Denis D (2008). MF498 [N-{[4-(5,9-dethoxy-6-oxo-6,8-dihydro-7H-yrrolo[3,4-g]quinolin-7-yl)- 3-methylbenzyl]sulfonyl}-2-(2-methoxyphenyl)acetamide], a selective E prostanoid receptor 4 antagonist, relieves joint inflammation and pain in rodent models of rheumatoid and osteoarthritis. *Journal of Pharmacology and Experimental Therapeutics*.

[75] Gomez PF, Pillinger MH, Attur M (2005). Resolution of inflammation: prostaglandin E2 dissociates nuclear trafficking of individual NF-*κ*B subunits (p65, p50) in stimulated rheumatoid synovial fibroblasts. *Journal of Immunology*.

[76] Stitham J, Midgett C, Martin KA, Hwa J (2011). Prostacyclin: an inflammatory paradox. *Frontiers in Pharmacology*.

[77] de Leval X, Hanson J, David JL, Masereel B, Pirotte B, Dogné JM (2004). New developments on thromboxane and prostacyclin modulators part 1: prostacyclin modulators. *Current Medicinal Chemistry*.

[78] Boie Y, Rushmore TH, Darmon-Goodwin A (1994). Cloning and expression of a cDNA for the human prostanoid IP receptor. *The Journal of Biological Chemistry*.

[79] Sugimoto Y, Hasumoto KY, Namba T (1994). Cloning and expression of a cDNA for mouse prostaglandin F receptor. *The Journal of Biological Chemistry*.

[80] Hébert RL, O’Connor T, Neville C, Burns KD, Laneuville O, Peterson LN (1998). Prostanoid signaling, localization, and expression of IP receptors in rat thick ascending limb cells. *American Journal of Physiology*.

[81] Hyunjung L, Dey SK (2002). Minireview: a novel pathway of prostacyclin signaling—hanging out with nuclear receptors. *Endocrinology*.

[82] Jaffar Z, Wan KS, Roberts K (2002). A key role for prostaglandin I2 in limiting lung mucosal Th2, but not Th1, responses to inhaled allergen. *Journal of Immunology*.

[83] Brodie MJ, Hensby CN, Parke A, Gordon D (1980). Is prostacyclin the major pro-inflammatory prostanoid in joint fluid?. *Life Sciences*.

[84] Dorris SL, Peebles RS (2012). PGI(2) as a regulator of inflammatory diseases. *Mediators of Inflammation*.

[85] Bley KR, Bhattacharya A, Daniels DV (2006). RO1138452 and RO3244794: characterization of structurally distinct, potent and selective IP (prostacyclin) receptor antagonists. *British Journal of Pharmacology*.

[86] Watanabe K (2002). Prostaglandin F synthase. *Prostaglandins and Other Lipid Mediators*.

[87] Sugimoto Y, Hasumoto KY, Namba T (1994). Cloning and expression of a cDNA for mouse prostaglandin F receptor. *The Journal of Biological Chemistry*.

[88] Pierce KL, Fujino H, Srinivasan D, Regan JW (1999). Activation of FP prostanoid receptor isoforms leads to rho-mediated changes in cell morphology and in the cell cytoskeleton. *The Journal of Biological Chemistry*.

[89] Nakahata K, Kinoshita H, Tokinaga Y (2006). Vasodilation mediated by inward rectifier K+ channels in cerebral microvessels of hypertensive and normotensive rats. *Anesthesia and Analgesia*.

[90] Takayama K, Yuhki KI, Ono K (2005). Thromboxane A2 and prostaglandin F2*α* mediate inflammatory tachycardia. *Nature Medicine*.

[91] Alexander CL, Miller SJ, Abel SR (2002). Prostaglandin analog treatment of glaucoma and ocular hypertension. *Annals of Pharmacotherapy*.

[92] Sugimoto Y, Yamasaki A, Segi E (1997). Failure of parturition in mice lacking the prostaglandin F receptor. *Science*.

[93] Breyer MD, Breyer RM (2001). G protein-coupled prostanoid receptors and the kidney. *Annual Review of Physiology*.

[94] Basu S (1999). Oxidative injury induced cyclooxygenase activation in experimental hepatotoxicity. *Biochemical and Biophysical Research Communications*.

[95] Basu S, Eriksson M (1998). Oxidative injury and survival during endotoxemia. *FEBS Letters*.

[96] Basu S, Whiteman M, Mattey DL, Halliwell B (2001). Raised levels of F2-isoprostanes and prostaglandin F2*α* in different rheumatic diseases. *Annals of the Rheumatic Diseases*.

